# Smart City Mobility Application—Gradient Boosting Trees for Mobility Prediction and Analysis Based on Crowdsourced Data

**DOI:** 10.3390/s150715974

**Published:** 2015-07-03

**Authors:** Ivana Semanjski, Sidharta Gautama

**Affiliations:** Department of Telecommunications and Information Processing, Gent University, St-Pietersnieuwstraat 41, Gent B-9000, Belgium; E-Mail: sidharta.gautama@ugent.be

**Keywords:** smart city, mobility management, modelling mobility decision making, gradient boosted trees, crowdsourcing

## Abstract

Mobility management represents one of the most important parts of the smart city concept. The way we travel, at what time of the day, for what purposes and with what transportation modes, have a pertinent impact on the overall quality of life in cities. To manage this process, detailed and comprehensive information on individuals’ behaviour is needed as well as effective feedback/communication channels. In this article, we explore the applicability of crowdsourced data for this purpose. We apply a gradient boosting trees algorithm to model individuals’ mobility decision making processes (particularly concerning what transportation mode they are likely to use). To accomplish this we rely on data collected from three sources: a dedicated smartphone application, a geographic information systems-based web interface and weather forecast data collected over a period of six months. The applicability of the developed model is seen as a potential platform for personalized mobility management in smart cities and a communication tool between the city (to steer the users towards more sustainable behaviour by additionally weighting preferred suggestions) and users (who can give feedback on the acceptability of the provided suggestions, by accepting or rejecting them, providing an additional input to the learning process).

## 1. Introduction

The development of information and communication technologies (ICT) has a big impact on peoples’ everyday life. This is not just evident in a way we communicate with each other, but also in the amount of information we produce daily, intentionally or unintentionally, and the potential this data brings to managing the urban environment. This potential is of particular research interest within the smart cities topic [1,2,3,4,5,6,7,8,9], and in this context, location acquisition technologies play an important basis for smart city applications [10,11].

When it comes to the mobility aspect of smart cities, location information acquisition is often supported by mobile phone data and, in the literature, there are some interesting examples of their use for extraction of origin-destination (OD) matrices [12,13,14,15,16] or derivation of travel behaviour information for model validation purposes [17,18]. The first attempts to provide personalized travel information services were made based on the analysis of data from public transport fare collection systems [19,20].

Nevertheless, little is known about the potential of crowdsourced data for smart city mobility management, especially in the context of personalized mobility services and the interactions between a city and its transportation system users. In this article we tackle this idea by using crowdsourced data from multiple sensors sources and a gradient boosting trees algorithm to model the personal mobility decision making process regarding the transportation mode selection for a set of given conditions (location, trip’s purpose, weather conditions, time of day, *etc.*). We see this as a potential platform for a city to steer the mobility of its inhabitants towards more sustainable behaviour by implementing the proposed model to provide personalised route suggestions for users via a dedicated smartphone application. Not only can the suggested approach enable city-individual communication, but it can provide users’ feedback where by accepting or rejecting personalised route suggestion the user evaluates the provided option.

## 2. Gradient Boosting Trees

To model the users’ decision making process regarding the transportation mode selection, we applied the gradient boosted trees (GBT) method. GBT is one of the most effective machine learning models for predictive analytics [21]. In general, it belongs to the family of decision tree learning methods which map observations about an item to conclusions about the item’s target value in a tree structure. Depending on the characteristics of the target value they can be used for regression (when the target variable is continuous) or classification (categorical target variable) purposes. In this context, further on we will focus just on the GBT classifier, as our target value is categorical (transportation mode).

### 2.1. Predictive Learning

The predictive learning problem consists of random explanatory variables (predictors) $x=\left\{x_{1},\;\ldots ,\;x_{n}\right\}$ and a random response variable y. By using a sample of known pairs of values ${\left\{y_{i},\;x_{i}\right\}}_{1}^{N}$ the goal is to obtain an estimate $\hat{F}\left(x\right)$, of the function $\tilde{F}\left(x\right)$ mapping x to y, that minimizes the value of loss function L(y, F(x)) over the joint distribution of all (x,y) pares Equation (1):

(1) $$\tilde{F}=\arg min_{F}E_{y,x}\;L\left(y,\;F\left(x\right)\right)=argmin_{F}E_{x}\left[E_{y}\left(L\left(y,F\left(x\right)\right)\right)|x\right]$$

Restricting the F(x) to be a member of parameterized class of functions  F(x,P), where $P=\left\{P_{1},\;P_{2},\ldots \;\right\}$ is a finite set of parameters whose joint values identify individual class members, changes the function optimization problem into parameter optimization problem Equation (2):

$$\tilde{F}\left(x\right)=F\left(x,\;\overset{ˇ}{P}\right)$$

where the value of parameter $\overset{ˇ}{P}$ is calculated as a sum of initial guess $p_{0}$ and all successive increments (“boosts”) ${\left\{p_{m}\right\}}_{1}^{M}$, each based on the sequence of proceeding steps Equation (3):

(3) $$\tilde{P}=\overset{M}{\underset{m=0}{\sum }}p_{m}$$

In general, boosting is used to increase the stability of the model [22], where for misclassified training events weights are increased (“boosted”) and a new tree is formed. To measure the successfulness of the prediction a separate, testing, data set is used. This procedure is repeated for new trees and the final score of the *m*th tree is the weighted sum of scores of the individual leaves.

### 2.2. GBT Algorithm

To model the users’ decision making regarding the selection of the transportation mode we use the GBT algorithm originally developed by Jerome H. Friedman [23]. In this algorithm the loss function $L\left(y_{k},\;F_{k}\left(x\right)\right)\;$ for the *k*-class problem Equation (4) is described as:

(4) $$L\left({\left\{y_{k},\;F_{k}\left(x\right)\right\}}_{1}^{K}\right)=-\overset{K}{\underset{k=1}{\sum }}y_{k}\log p_{k}\left(x\right)$$

where $y_{k}=1\;\left(class=k\right)\in \left\{0,1\right\}$ and $p_{k}\left(x\right)=\mathrm{Pr}\left(y_{k}=1\text{|}x\right)$. In addition, the logistic transformation is used to the predicted values before computing residuals, scaled to a probability scale where each tree has J terminal nodes with corresponding regions ${\left\{R_{jkm}\right\}}_{j=1}^{J}\;$ and to compute the final classifications Equation (5):

(5) $$\gamma_{jkm}=\frac{K-1}{K}\frac{\sum_{x_{i}\epsilon R_{jkm}}{\tilde{y}}_{ik}}{\sum_{x_{i}\epsilon R_{jkm}}\left|{\tilde{y}}_{ik}\right|\left(1-\left|{\tilde{y}}_{ik}\right|\right)}$$

The steps of the used GBT Algorithm are given below and for more details we refer the reader to the source publications [23,24], whereas the more general overview of the decision threes and the GBT can be found in literature [25,26,27].

**GBT Algorithm**$F_{k0}\left(x\right)=0,\;k=1,\;K$For m = 1 to M do:$$p_{k}\left(x\right)=\exp\left(F_{k}\left(x\right)\right)/\overset{K}{\underset{l=1}{\sum }}\text{exp}(F_{l}\left(x\right),\;k=1,\;K$$For k=1  to K do:$${\tilde{y}}_{ik}=y_{ik}-p_{k}\left(x\right),\;i=1,\;N$$$${\left\{R_{jkm}\right\}}_{j1}^{J}=j\;\text{terminal node}\;tree\;\left({\left\{{\tilde{y}}_{ik},x_{i}\right\}}_{1}^{N}\right)$$$$\gamma_{jkm}=\frac{K-1}{K}\frac{\sum_{x_{i}\epsilon R_{jkm}}{\tilde{y}}_{ik}}{\sum_{x_{i}\epsilon R_{jkm}}\left|{\tilde{y}}_{ik}\right|\left(1-\left|{\tilde{y}}_{ik}\right|\right)},\;j=1,\;J$$$$F_{km}\left(x\right)=F_{k,\;m-1}\left(x\right)+\;\overset{J}{\underset{j=1}{\sum }}\gamma_{jkm}1\left(x\epsilon R_{jkm}\right)$$endForendForEnd Algorithm

### 2.3. Data Collection

As input values for the GBT algorithm we rely on three data sources:

(a) a dedicated smartphone application [28] with active logging (users can provide input by actively tracking their routes and defining the trip’s purpose and transportation mode used) or the application can be set in a passive mode (whereby the tracks are passively logged and automatically segmented into trips with a separate IDs and transportation mode detected based on the Google Activity recognition API [29]).
(b) a dedicated geographic information system (GIS) web interface [30] where users can register and give basic information about their mobility behaviour as routes often used, trip purposes, *etc*.
(c) a weather forecast API [31] that provides information about the weather conditions at the requested location.

**Figure 1:**
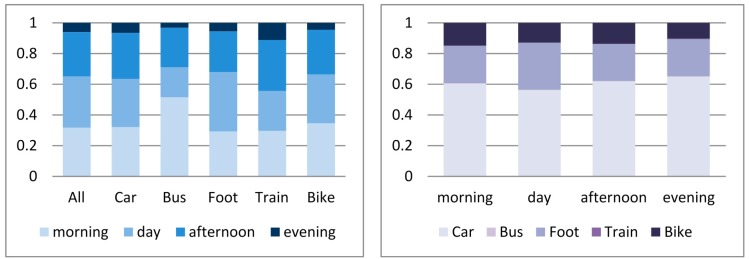
Distribution of trips (kilometres) made by mode (**Left**) and time of day (**Right**).

Based on these data sources over 4000 trips were recorded during a period of six months (Table 1). Considering the distribution of the recorded trips, the least of them were made during the evening hours (after 19 h), whereas in general the most kilometres were travelled by car, followed by foot and bike (Figure 1).

Based on these three data sources a set of predictor variables is created in order to model the users’ decision making process when it comes to the selection of transportation mode for a given trip in a given circumstances. Table 2 shows the full list of variables with acronyms and description.

**Table 1:** Data set descriptive information.

Sample Size	Km Travelled	Time Span	Trips Recorded
292 users	37,121	180 days	4005

**Table 2:** Variables used for the modelling transportation mode selection decision process.

Variable	Acronym	Description	Source
User’s ID	userid	Unique identifier of the user/device	a, b
Trip’s ID	tripid	Unique identifier of the trip	a, b
Trip’s start time	starttime	Year, month, day, hour, minute and second when trip started	a
Trip’s stop time	stoptime	Year, month, day, hour, minute and second when trip ended	a
Trip’s start location	startpoint	Geographic location of the trip’s origin point	a
Trip’s end location	endpoint	Geographic location of the trip’s destination point	a
Distance	distance	Distance between trip’s origin and destination points measured in kilometres	a
Transportation mode	transportmode	Transportation mode used for the trip	a
Trip’s purpose	purpose	The purpose of the trip made (go to work, shopping, recreation, school…)	a, b
Working day identification	week day	Boolean value that indicates if the day when trip started is a working day	a
Holiday identification	weekend	Boolean value that indicates if the day when trip started is a holiday or weekend	a
Average temperature	TemperatureAvgC	Average temperature for the trips location measured in Celsius degrees	c
Dew point	DewpointAvgC	The average temperature at which the water vapour in a sample of air at constant barometric pressure condenses into liquid water at the same rate at which it evaporates, measured in Celsius degrees	c
Humidity	HumidityAvg	The average amount of water vapour in the air, measured in hectopascals	c
Wind speed	WindSpeedAvgKMH	Average wind speed, measured in kilometres per hour	c
Precipitation	PrecipitationSumCM	Sum of precipitation during a day when trip was made, measured in centimetres	c

## 3. Modelling the Individual’s Mobility Decision Making from the Crowdsourced Data

For the purpose of modelling the individuals’ mobility decision making process we selected an individual (ID = 23), who logged 311 trips. These trips were made by three transportation modes: car, foot and bike. The goal of the GBT algorithm was to successfully learn which transportation mode the user is most likely to use for a given purpose, weather conditions, origin and destination location pairs, trip distance, starting time of the trip and in regard to the working day or holiday/weekend condition. The learning process is based on the previous behaviour of the user (training data set) and results are then compared to the test data set (separate data set that also contains information on the user behaviour, but was not used for the learning process) in order evaluate the success of the learning results.

### 3.1. Optimal Number of Trees

**Figure 2:**
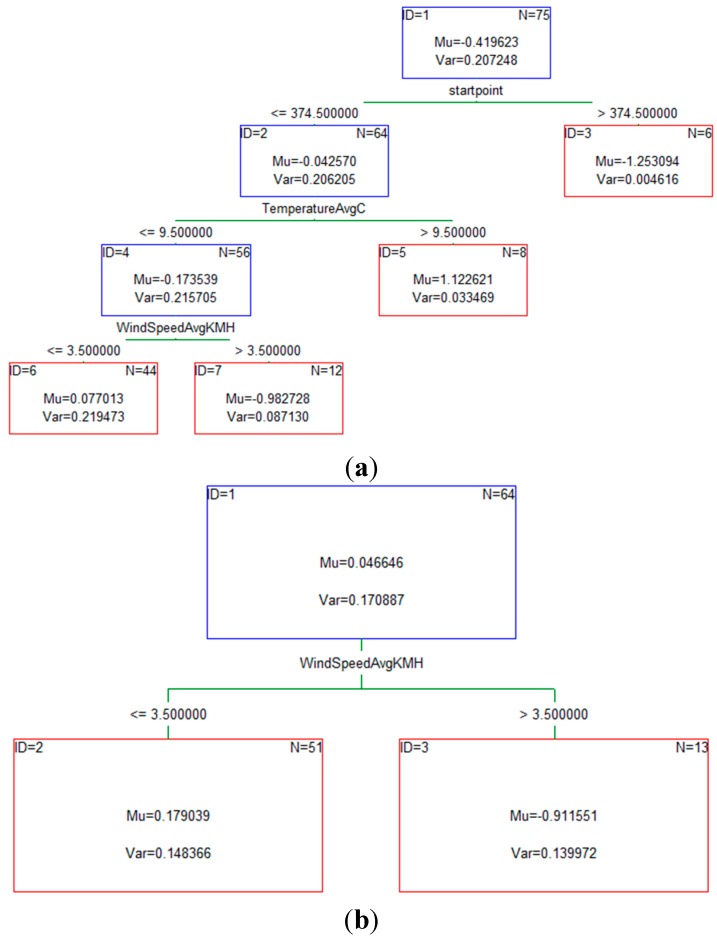

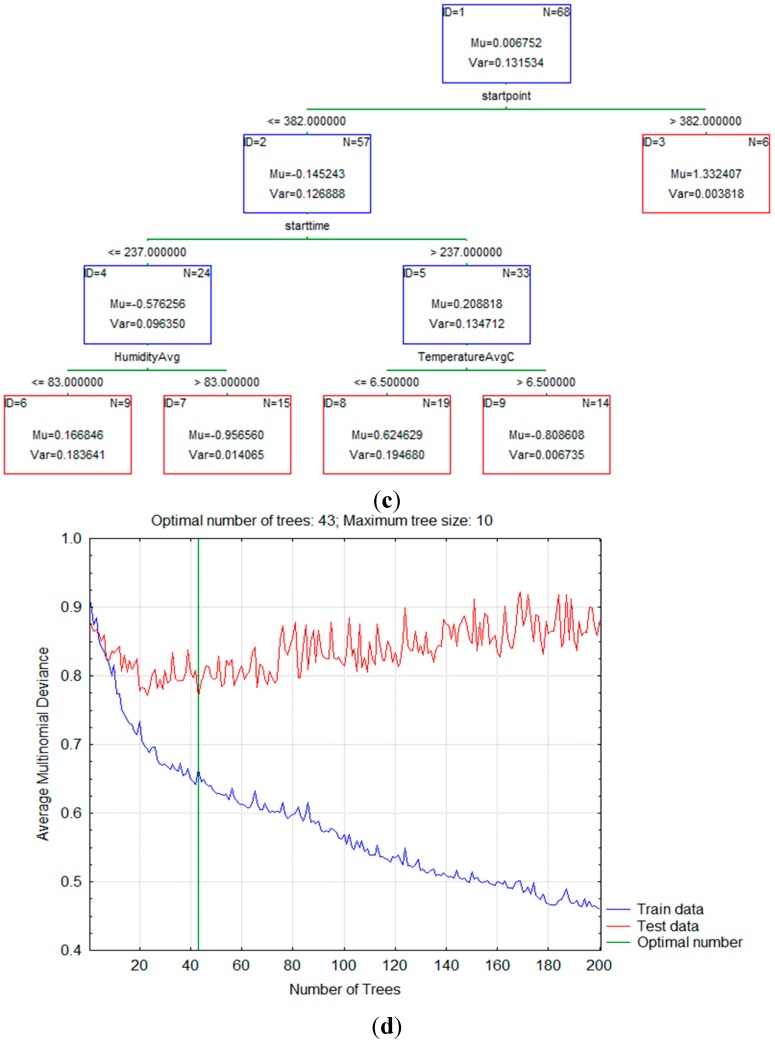
(**a**) An example of the simple tree for the transportation mode bike; (**b**) An example of the simple tree for the transportation mode walk; (**c**) An example of the simple tree for the transportation mode car; (**d**) Average multinomial deviance for boosted trees.

The first step in building a model was to compute a sequence of (very) simple decision trees, where each successive tree was built for the prediction residuals of the preceding tree. Figure 2 shows examples of some of those simple decision trees that were used in the building process.

As more and more trees were added to the model, the average squared error function for the training data (from which the respective trees were estimated) decreased. This clearly showed the improvement of the learning process as the model was able to learn from the errors of previous trees and make more accurate predictions regarding the transportation mode that the individual will use. Based on the average squared error value we were able to estimate the optimal number of trees as it clearly marked the point where the smallest error for the testing data occurred. Table 3 shows values of standard errors for both test and train data set.

**Table 3:** The GBT classification’s standard error.

	Standard Error
Train	0.028980
Test	0.056912

### 3.2. Predictors Importance

**Figure 3:**
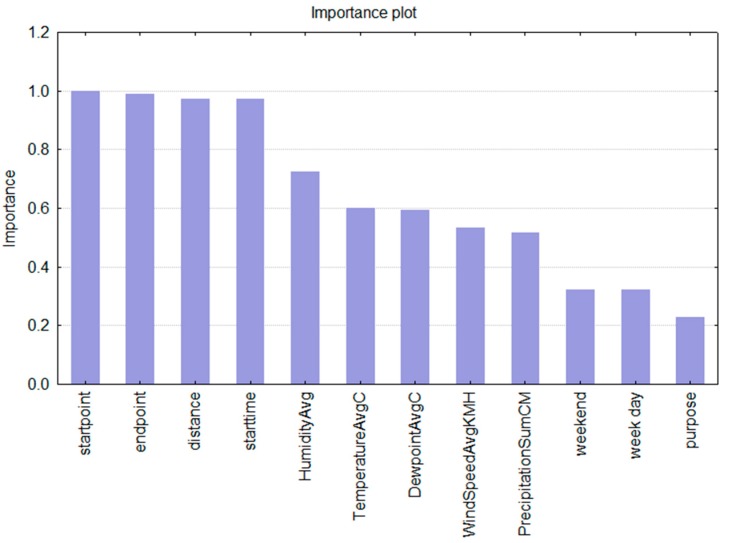
Predictor variables importance plot.

Next to the standard error values, which serve as an indication of the overall model’s quality an pertinent insight into the decision making process is the calculated importance of predictors (Figure 3). The predictor importance value shows what predictors influenced the decision about the selection of the transportation mode the most. One can see that the decision about the transportation mode for any given trip and the selected individual is mainly based upon the information on the location, followed by the indication of the starting time for the trip. Correlation analysis gave the highest (and statistically significant) value (0.328828) for evening hours, meaning that this factor has a high influence on the decision about what transportation mode to choose. On the other hand, for the city, this can indicate that in the evening hours fewer public transportation lines, at certain locations, limit the mobility options and therefore result in a less sustainable mobility behaviour (usage of the car).

Regarding the weather-related predictors, the humidity and the dew point have the highest importance in the decision making process of a given individual. In addition, the decision is the least influenced by the information on working/not working day and the trip’s purpose.

### 3.3. Classification Matrix

**Figure 4:**
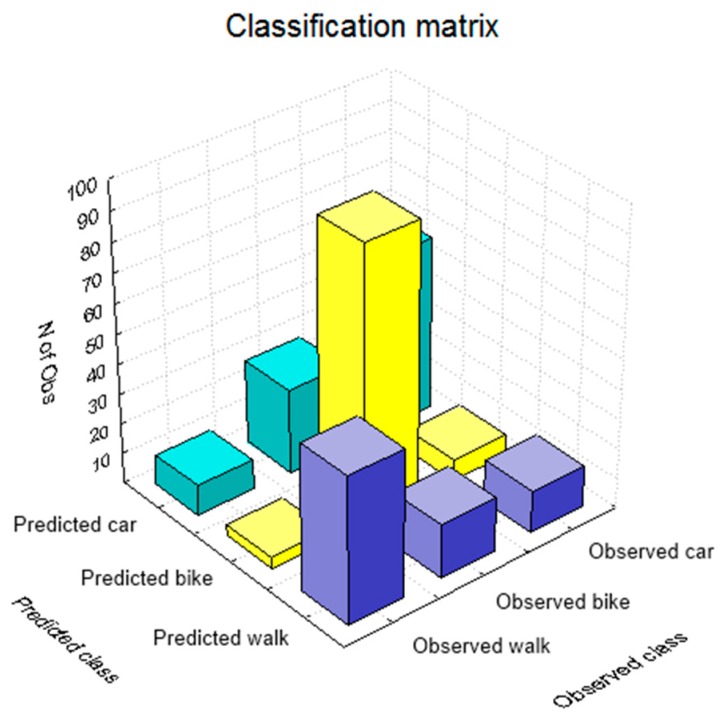
Classification matrix histogram.

The classification matrix gives an overview of correctly classified and misclassified values or when the built model successfully predicted which transportation mode the user will select and when not. Figure 4 shows the histogram of the classification matrix, where the highest values on the diagonal of the histogram mean that these transportation modes were correctly classified or that the boosting trees were able to correctly model the user’s decision making process from the given dataset. Table 4 gives a more detailed overview of the classification results. The overall success of the boosted trees model to correctly recognize the transportation mode that user will select in the certain circumstances is 73%. The highest success was obtained for the transportation mode walk, followed by car and bike, while the user made the most of the trips by bike. The highest misclassification occurred between the car and the bike (28 trips), which corresponds to the 20% of all bike trips and the lowest among the walk and bike (4 trips or 6% of all walk trips).

**Table 4:** Classification matrix details.

Observed		Predicted Walk	Predicted Bike	Predicted Car	Row Total
**Count**	walk	49	4	10	63
**Column Percentage**		60.49%	3.88%	10.42%	
**Row Percentage**		77.78%	6.35%	15.87%	
**Total Percentage**		17.50%	1.43%	3.57%	22.50%
**Count**	bike	18	92	28	138
**Column Percentage**		22.22%	89.32%	29.17%	
**Row Percentage**		13.04%	66.67%	20.29%	
**Total Percentage**		6.43%	32.86%	10.00%	49.29%
**Count**	car	14	7	58	79
**Column Percentage**		17.28%	6.80%	60.42%	
**Row Percentage**		17.72%	8.86%	73.42%	
**Total Percentage**		5.00%	2.50%	20.71%	28.21%
**Count**	All Groups	81	103	96	280
**Total Percentage**		28.93%	36.79%	34.29%	

### 3.4. Transportation Mode Predictions

Table 5 shows a sample of the boosting trees predictions for the test dataset. Next to the more detailed insight into the misclassified values the predictions give a score that each transportation mode obtained for a given trip. This way, one can evaluate what transportation mode would be the second option to the user for a given trip in a case the first option was temporary unavailable, or it can also indicate what option for a given trip and the given circumstances is the least favourable by the user (next to the transportation modes that were not used during the data collection period by this individual). Here, the highest potential for the city to impact the user’s transportation mode selection decision process is seen.

**Table 5:** Boosting tree predictions.

	Observed Value	Predicted Value	Probability for Walk	Probability for Bike	Probability for Car
259	bike	bike	0.0234	0.934625	0.041975
261	bike	bike	0.046108	0.912823	0.041068
262	bike	bike	0.030055	0.923156	0.046789
263	bike	bike	0.106958	0.842734	0.050308
264	bike	bike	0.062376	0.880292	0.057332
265	bike	bike	0.023703	0.951022	0.025275
266	car	car	0.102277	0.159174	0.738549
267	bike	bike	0.027343	0.930179	0.042478
268	car	car	0.103929	0.189645	0.706426
269	car	car	0.077642	0.323658	0.598699
270	bike	bike	0.025509	0.934864	0.039628
271	bike	bike	0.020835	0.945525	0.03364
272	bike	bike	0.085094	0.68833	0.226576
273	bike	bike	0.04176	0.852439	0.105802
274	bike	bike	0.086507	0.673364	0.240129
275	bike	car	0.155593	0.40531	0.439097
276	bike	bike	0.076748	0.767849	0.155403
277	bike	bike	0.06233	0.887722	0.049949
278	bike	bike	0.055385	0.776124	0.168491
279	car	car	0.055508	0.062741	0.881751
280	walk	car	0.332184	0.208863	0.458953
281	bike	bike	0.05002	0.900994	0.048987
282	bike	bike	0.159287	0.685904	0.154809
283	bike	bike	0.086926	0.579401	0.333673
285	bike	bike	0.073421	0.628132	0.298447
286	bike	bike	0.028764	0.913341	0.057895
287	bike	bike	0.026671	0.946023	0.027305
289	bike	bike	0.067302	0.710574	0.222125
290	car	car	0.054037	0.062914	0.883049
291	car	car	0.031149	0.047442	0.92141
292	car	car	0.031286	0.081858	0.886856
293	bike	bike	0.171193	0.451166	0.377641
295	car	car	0.096279	0.171312	0.732409
296	bike	bike	0.04745	0.918952	0.033598
297	car	car	0.238833	0.128685	0.632482
298	car	car	0.099837	0.062583	0.83758
300	bike	bike	0.121236	0.472548	0.406216
301	bike	bike	0.024016	0.933427	0.042557
303	car	car	0.059362	0.103285	0.837353
304	car	car	0.096316	0.069332	0.834352
305	car	car	0.040802	0.089445	0.869753
306	car	car	0.057023	0.084437	0.85854
307	car	car	0.040802	0.089445	0.869753
308	car	car	0.041736	0.051064	0.9072
310	car	car	0.027506	0.06945	0.903044

For example, when scores for the two transportation modes are quite close, the city can favour the more sustainable one and give it priority or based on the starting and ending location add additional score/weight to the transportation mode that it wants to promote in that area (e.g., if there is a bike highway infrastructure that corresponds to the user’s trip location, and city wants to promote its usage). This can be communicated to the user in the order route suggestions appear when requested and based on the knowledge gained, from the decision making process model, the city can provide automatized and personalized route guidance for each user to steer their behaviour towards the more desirable one in the sustainability sense.

## 4. Conclusions

In this article we successfully modelled, based on the detailed behavioural data, what transportation mode an individual is likely to use in a given context. To do this we applied gradient boosted trees and crowdsourced data from three sources (a smartphone application, a GIS-based web interface, and weather sensors). These data were divided into two data sets—training and test—and the overall success of the suggested model was 73%. It should be noted that the expected results depend on the quality of the input data, therefore advances in the location detection precision, automation of transportation mode detection for passively collected data and trip segmentation can positively influence the quality of decision making model. With this in mind, our future research will be focused on trip chaining and the detection of multimodal combinations to be used and suggested to the user.

The potential applications of the developed model include provision of personalized services as well as personalized routing suggestions to users via a dedicated smartphone application. This is particularly interesting in the context of steering users’ mobility behaviour towards the more sustainable one as the city is able, in the ranking/scoring suggestions, to weight the preferred ones. Also, by using the personalised routing the city can gradually impact the overall mobility behaviour of its citizens by making it more synchronized. For example, instead applying the “one-for-all” solutions (as redirecting all users towards the less crowded streets, and in that sense solving the local problem, whereas in the sense of the connected network impact, this action usually just relocates the traffic jams towards the new location), the combined small changes made by individuals could be managed to have pertinent and balanced joint impact. Further on, the suggested model can be used as a smart city mobility management platform and communication tool between policy makers and citizens, where via suggestions city can communicate more preferable routes and users can, by accepting or rejecting the suggestions, provide feedback on the personalized results and route preferences. In addition, city can gains insights on the usage of its network infrastructure as well as manage traffic flows in incident situations by providing personalized alternative routes is such cases. For a user, the developed model can be seen as a filtering tool whereby for a given trip he will not need to consult several search engines (e.g., national rail company for train routes, local public transportation company for bus routes, Google maps for pedestrian routes, *etc.*) and route suggestions can be searched and displayed on one place in line with his personal preferences.
